# Hypertrophic Olivary Degeneration and Palatal or Oculopalatal Tremor

**DOI:** 10.3389/fneur.2017.00302

**Published:** 2017-06-29

**Authors:** Caroline Tilikete, Virginie Desestret

**Affiliations:** ^1^Neuro-Ophthalmology and Neurocognition, Hôpital Neurologique Pierre Wertheimer, Hospices Civils de Lyon, Bron, France; ^2^Lyon I University, Lyon, France; ^3^ImpAct Team, CRNL INSERM U1028 CNRS UMR5292, Bron, France; ^4^SynatAc Team, Institut NeuroMyogène INSERM U1217/UMR CRS 5310, Lyon, France

**Keywords:** symptomatic palatal tremor, progressive ataxia and palatal tremor, pendular nystagmus, hypertrophic degeneration of inferior olive, dentato–olivary pathway, Guillain–Mollaret triangle

## Abstract

Hypertrophic degeneration of the inferior olive is mainly observed in patients developing palatal tremor (PT) or oculopalatal tremor (OPT). This syndrome manifests as a synchronous tremor of the palate (PT) and/or eyes (OPT) that may also involve other muscles from the branchial arches. It is associated with hypertrophic inferior olivary degeneration that is characterized by enlarged and vacuolated neurons, increased number and size of astrocytes, severe fibrillary gliosis, and demyelination. It appears on MRI as an increased T2/FLAIR signal intensity and enlargement of the inferior olive. There are two main conditions in which hypertrophic degeneration of the inferior olive occurs. The most frequent, studied, and reported condition is the development of PT/OPT and hypertrophic degeneration of the inferior olive in the weeks or months following a structural brainstem or cerebellar lesion. This “symptomatic” condition requires a destructive lesion in the Guillain–Mollaret pathway, which spans from the contralateral dentate nucleus *via* the brachium conjunctivum and the ipsilateral central tegmental tract innervating the inferior olive. The most frequent etiologies of destructive lesion are stroke (hemorrhagic more often than ischemic), brain trauma, brainstem tumors, and surgical or gamma knife treatment of brainstem cavernoma. The most accepted explanation for this symptomatic PT/OPT is that denervated olivary neurons released from inhibitory inputs enlarge and develop sustained synchronized oscillations. The cerebellum then modulates/accentuates this signal resulting in abnormal motor output in the branchial arches. In a second condition, PT/OPT and progressive cerebellar ataxia occurs in patients without structural brainstem or cerebellar lesion, other than cerebellar atrophy. This syndrome of progressive ataxia and palatal tremor may be sporadic or familial. In the familial form, where hypertrophic degeneration of the inferior olive may not occur (or not reported), the main reported etiologies are Alexander disease, polymerase gamma mutation, and spinocerebellar ataxia type 20. Whether or not these are associated with specific degeneration of the dentato–olivary pathway remain to be determined. The most symptomatic consequence of OPT is eye oscillations. Therapeutic trials suggest gabapentin or memantine as valuable drugs to treat eye oscillations in OPT.

## Introduction

The terminology and the nosology of hypertrophic inferior olive degeneration and palatal tremor (PT) or oculopalatal tremor (OPT) has evolved over time and needs some clarification. Unilateral or bilateral hypertrophic olivary degeneration (HOD) in the medulla oblongata was first anatomically described in late nineteenth century (1). At the same time, literature focused on the observation of rhythmic PT (2) using different terms such as palatal nystagmus, palatal myoclonus, or palatal myorhythmia. It was finally classified among tremors in 1990 (3). PT is often associated with synchronous eye oscillations and such cases are termed OPT. It can also be associated with synchronous movements of the larynx, pharynx, diaphragm, and facial muscles. PT or OPT has been described in association with the anatomical observation of HOD (4). HOD was later demonstrated on MRI, where it appears as an increased T2/FLAIR signal intensity and enlargement of the inferior olive (5–7). This unique degeneration of the inferior olive most frequently develops weeks or months (8, 9) secondary to a lesion within the dentato–olivary pathway (10), originally referred to as the Guillain–Mollaret triangle (11). The lesion is most often a hemorrhagic stroke.

In 1990, Deuschl et al. suggested differentiating symptomatic PT, developing secondary to brainstem or cerebellar lesions, from essential PT (EPT) for which there is no evidence of a structural lesion (12). Patients with EPT usually have objective ear click, which is less frequent (8%) in the symptomatic form. Involvement of the tensor veli palatini muscle in EPT and of the levator veli palatini muscle in symptomatic PT might explain this clinical difference (3). However, those with symptomatic PT may also experience ear click; to distinguish forms it is of note that EPT patients neither show involvement of eye and other muscles nor evidence of structural abnormalities of the inferior olive (13). Furthermore, the etiology of EPT is heterogeneous with a considerable proportion of psychogenic cases (14) and may disappear over time (15). EPT is therefore a different disease without HOD and does not concern this review; below, PT refers to the symptomatic form.

Later on, Sperling and Herrmann (6) and then Samuel et al. (13) described a syndrome of progressive ataxia and palatal tremor (PAPT). Some of them disclose OPT. In these cases, ataxia progresses and is not the result of a monophasic illness. Sporadic and familial forms of PAPT are described. There is no visible structural causative lesion on the dentato–olivary pathway, but HOD on MRI is present in most cases. Although a specific lesion of the dendato–olivary pathway is not yet identified, PAPT could be considered as a subgroup of symptomatic PT or OPT and will therefore be described in this review.

## Clinical Features of PT and OPT

The first observations of synchronous rhythmical movement of the eye and palate were published 150 years ago (2). Since then, different publications have reported the clinical features of this abnormal palatal and eye movement (11, 16, 17).

Symptomatic PT is characterized by involuntary movements of the soft palate and pharynx, due to rhythmic contraction of the levator veli palatine (8, 16) (Video S1 in Supplementary Material). The movements are most commonly bilateral and symmetrical (18). In this case, the soft palate is contracted superiorly and posteriorly along with the uvula with synchronous closing of the pharynx (8). Sometimes the movement can be unilateral, the palate and uvula then being drawn to one side (17, 19). The movements are continuous, the rhythm being most frequently between 100 and 160/min (or 1.5–3 Hz) and persist during sleep (3). Patients with symptomatic PT very rarely complain of ear click (3, 18).

**Figure 1:**
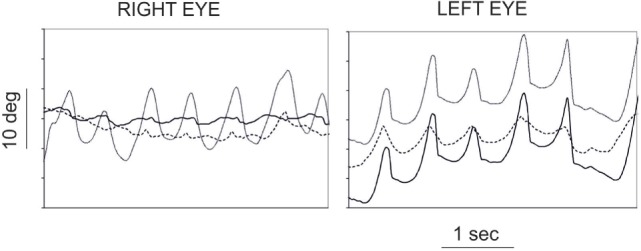
Eye position (in degrees) traces according to time (in seconds) for right (left panel) and left (right panel) eye in an oculopalatal tremor patient. Continuous line: horizontal position, discontinuous line: vertical position, and dotted gray line: torsional position. Adapted from Ref. (24).

Oculopalatal tremor refers to the synchronous combination of PT and pendular nystagmus. Pendular nystagmus is found to be present in 30% of symptomatic PT (3), probably less frequently in case of PAPT [4 out of 28 cases in Samuel et al. (13)]. In series of patients with pendular nystagmus, up to 18% of those with HOD do not develop PT (20, 21). Patients have mainly vertical pendular oscillations of the eyes with varied combinations of torsional and horizontal components (21–24) (Video S2 in Supplementary Material). The nystagmus can sometimes take the form of convergent–divergent nystagmus (20, 25). This pendular nystagmus is of quite large mean amplitude (8°), high peak velocity (16°/s), and demonstrates irregularity (24) (Figure 1). It is most frequently asymmetric and dissociated in direction in the two eyes (24). While PT is mostly asymptomatic, patients with OPT complain of disturbing oscillopsia, decreased visual acuity, with deterioration of vision-specific health-related quality of life (24, 26). Other than the observed synchrony, attempts have been made to relate characteristics of the nystagmus to the associated palatal movements (22) and to the side of HOD, but the randomness of the directions, waveforms, as well as disconjugacy of nystagmus could just reflect randomly formed couplings in inferior olivary neurons (27). Furthermore, the other associated ocular motor deficit secondary to the brainstem lesion may contribute to disconjugacy of the nystagmus (28).

Other synchronous movements can be associated with palatal myoclonus, most frequently involving muscles of the gill arches: the face, the tongue, the floor of the mouth, the pharynx, the larynx, and the diaphragm (29) (Videos S1 and S2 in Supplementary Material). In some rare cases, skeletal muscle tremor, mainly of the upper limbs, may be associated (30–32). Some cases of OPT, secondary to lesion of the dentato–olivary pathway, present with focal or generalized dystonia, constituting a variant of OPT (33).

## Etiologies

### Symptomatic PT and OPT

**Figure 2:**
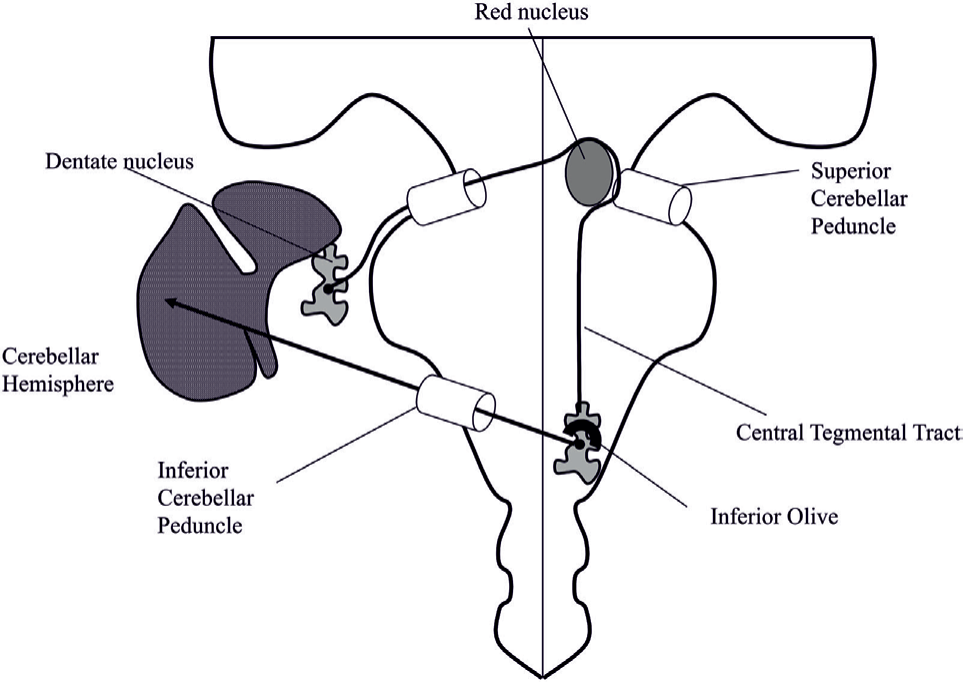
Schematic representation of the Guillain–Mollaret triangle. The pathway coming from the contralateral dentate nucleus, through the contralateral brachium conjunctivum crosses the midline, turns around the ipsilateral red nucleus, and descends in the ipsilateral central tegmental tract to the inferior olive. Adapted from Ref. (36).

According to the earliest described cases (2, 4, 11, 16, 17), the most common form of PT/OPT is secondary to a monophasic structural lesion of the brainstem or the cerebellum. The topography of the lesion involves the dentato–olivary pathway, part of the Guillain–Mollaret triangle (11), i.e., the pathway coming from the contralateral dentate nucleus, through the contralateral brachium conjunctivum crossing the midline, turning around the ipsilateral red nucleus, and descending in the ipsilateral central tegmental tract to the inferior olive (Figure 2). Central tegmental tract lesions are the most frequent and seem to be more specifically associated with OPT compared to lesions of dentate nuclei/brachium conjunctivum where only PT is observed (21, 24, 34). In these cases of symptomatic PT or OPT, the condition develops at least 1 month and up to 8 years (median between 10 and 11 months) after the occurrence of the presumed anatomical lesion (9, 13). Symptomatic PT becomes increasingly intense, reaching a peak between 5 and 24 months after lesion (35). Once established, PT or OPT persists for life, with the exception of a few patients in whom PT or OPT is reported to have disappeared completely after many years, although MRI show persistent signal change in the inferior olivary nucleus (21) (Video S3 in Supplementary Material).

The most frequent etiology of structural brainstem or cerebellar lesion is vascular and more often hemorrhagic than ischemic (11, 37). Other etiologies include brain trauma, brainstem tumors, surgical or gamma knife removal of brainstem cavernoma (38), multiple sclerosis (MS), and a broad range of other unspecific lesions [see Table 3 in Samuel et al. (13)]. It is assumed that, to be causative, this primary lesion has to be destructive, a condition that is most easily satisfied by vascular, neurosurgical, or gamma knife lesions (19).

The MS cases might be further discussed. MS has been identified as a common cause of OPT, from 3 (12) to 10% (13). Mostly historical articles report cases of OPT or PT secondary to MS (39, 40). However, in MS, pendular nystagmus is much more often observed without OPT (24, 41, 42). This pendular nystagmus is of small amplitude (1°), low mean peak velocity (6°/s), high mean frequency (4–6 Hz), and is highly regular, like a sine wave (24, 41). Although confused with OPT (42), there is neither an associated PT nor HOD on MRI (24). It may be added that in historical neuropathological cases, almost all lesions are vascular in nature (11). The relatively high proportion of reported MS in PT and OPT might therefore have been overestimated. Indeed, a review of historical cases finds that they do not meet the current clinical criteria for diagnosis of MS or OPT, and neuroimaging or pathology was lacking (40, 43). Some cases seem to correspond to pendular nystagmus associated with MS, other to brainstem hemorrhage, sporadic, or familial PAPT. A notable exception is the report of two patients with clinical, biological, and MRI criteria for MS, developing OPT associated with HOD on MRI (44, 45). Although the second case was complex with history of posterior fossa tumor and radiation therapy (45), these are the only convincing observations of OPT in MS.

According to the topography of the structural lesion, other neurological manifestations may be observed in association with OPT. Patients frequently present contralateral hemiplegia, contralateral hemi-hypoesthesia or spinothalamic syndrome, ipsilateral facial palsy, ipsilateral kinetic cerebellar syndrome (24). In the case of unilateral cerebellar signs, pendular nystagmus is more pronounced in the eye on the affected side (3). Patients also frequently have a deficit in the horizontal eye movement, including fascicular abducens nerve palsy, internuclear ophthalmoplegia, one and a half syndrome, nuclear abducens syndrome (nuclear VI), or horizontal saccadic palsy (24, 41). Central vestibular manifestations have also been reported in association to OPT (46). These manifestations usually result from the primary lesion and present as a monophasic event.

Delayed and progressive worsening of extremity and gait cerebellar ataxia associated with OPT, secondary to identified structural etiologies (stroke; cavernoma; tumor and radiation therapy; subarachnoid hemorrhage; brain trauma) has also been reported (36, 47, 48). The mechanisms of OPT with delayed ataxia following brainstem lesion is not understood, although it seems to occur with larger and bilateral acute brainstem lesions (47). Hemosiderin deposition has been suggested (48), but it cannot explain the cases observed in brainstem tumors and radiotherapy. Delayed ataxia or movement disorder following a monophasic structural lesion without OPT has been reported (49, 50), which could suggest that not all progressive disorders arise from primary neurodegenerative processes (13).

### Progressive Ataxia and PT

In 1985, Sperling and Herrmann (6) suggested to distinguish a syndrome associating PT, HOD, and progressive cerebellar ataxia. This entity was reported again (51), and the syndrome of progressive ataxia and palatal tremor (PAPT) was more precisely defined by Samuel et al. (13). The authors suggested differentiating sporadic PAPT from familial forms of PAPT. None of the patients have structural brainstem or cerebellar lesion, but cerebellar ataxia and cerebellar atrophy on MRI progress over years. These cases might correspond to the “degenerative” etiology suggested in older reports [see review in Samuel et al. (13)].

#### Sporadic PAPT

In sporadic PAPT, other than gait, trunk and limb ataxia, dysarthria, non-specific cerebellar ocular motor dysfunction is observed, such as gaze-evoked nystagmus, jerk vertical nystagmus, hypermetric saccades, and saccadic pursuit (13). All reported patients present PT. Four out of 28 patients reviewed in Samuel et al. (13) have OPT and 2 internuclear ophthalmoplegia, which indicate brainstem involvement. Patients often complain of poor vision due to oscillopsia or diplopia. Hearing loss seems to be quite frequently associated with sporadic PAPT (in four out of six patients). Other neurological manifestations are not specific. The cerebellar ataxia may precede or follow the occurrence of PT (52). Almost all patients show abnormal bilateral signal and/or HOD on MRI. There is no single theory unifying etiologies of sporadic PAPT, although some of them might be due to polymerase gamma (POLG) mutation (53, 54).

In older reports, PT and HOD has also been reported in other degenerative neurological disorders such as pathologically proven progressive supranuclear palsy (55, 56) and other undetermined neurodegenerative diseases (57). The nosology of these cases, presenting with progressive neurological deficit, other than cerebellar ataxia, needs to be clarified.

#### Familial PAPT

Familial PAPT is more complex than sporadic PAPT and may include a variety of etiologies. They are associated with marked brainstem and cervical cord atrophy with corticospinal tract findings, and the olivary MRI abnormalities may be lacking (13). Three main known etiologies may be considered: Alexander disease, POLG mutation, and spinocerebellar ataxia type 20 (SCA20).

Alexander disease is one of the most reported known etiologies of familial progressive neurological disorder associated with PT (57–61). Alexander disease is a leukodystrophy, that is pathologically characterized by the presence of Rosenthal fibers, and that is caused by mutations in the gene encoding glial fibrillary acidic protein on chromosome 17q21 (62) and present as a progressive neurological disorder that can occur in an infantile, juvenile, or adult form (59). It usually results from *de novo* mutations, with autosomal dominant inheritance in future generations (59). In juvenile and adult forms, the patients exhibit palatal myoclonus, spastic tetraparesia, mild cerebellar dysfunction, and associated ocular motor abnormalities (60). There is no description of HOD in large series of adult-onset Alexander disease (63), but one recent case with a phenotype of PAPT presented inferior olive hypertrophia (64). In only one case, associated “ocular myoclonus” was described (60).

Recent observations of PT or OPT with HOD, or HOD without clinical manifestations of PT or OPT have been reported in association with POLG mutation (53, 54, 65). Mutations of the mitochondrial DNA (mtDNA) encoded by the POLG gene are an important cause of pediatric and adult-onset mitochondrial disease. In adults, they are associated with multiple mtDNA deletions leading to a wide spectrum of dominant and recessive progressive neurological disorders, often described as syndromes, such as progressive external ophthalmoplegia, Alpers syndrome, sensory ataxic neuropathy, dysarthria and ophthalmoparesis (65, 66). POLG mutation should also be considered in patients with PAPT or progressive ataxia with inferior olive hypersignal (54), even in sporadic cases, and even without other frequently associated neurological signs such as sensory neuronopathy associated with weakness of ocular, pharyngeal, axial, and/or limb muscles (66).

Autosomal dominant SCA20 is a rare spinocerebellar ataxia characterized by a slowly progressive ataxia and dysarthria; two-thirds of those affected also display PT (“myoclonus”) with increased inferior olivary T2 signal (67). In these patients, CT scan shows dentate calcification, without concomitant pallidal calcification. The locus of genetic mutation overlaps that of spinocerebellar ataxia type 5 on chromosome 11, but the phenotypes are very different (68). More recently, a single case of adult-onset GM2-gangliosidosis type II (Sandhoff disease) presenting PT and cerebellar ataxia has been reported, although inferior olive signal was not described (69).

### Toxic HOD

There are few reports of reversible inferior olive MRI hypersignal among diffuse MRI changes associated with toxic-induced encephalopathy, such as metronidazole (70). Although none of them was associated with the clinical syndrome of PT or OPT, toxic lesions have a predilection for dentate nuclei and brainstem tegmentum, suggesting reversible lesion of the Guillain–Mollaret triangle (70). One case of reversible PT induced by fluoxetine has been reported, although HOD on MRI is not mentioned (71).

## Neuropathology of the Degenerative Hypertrophic Inferior Olivary Nucleus

**Figure 3:**
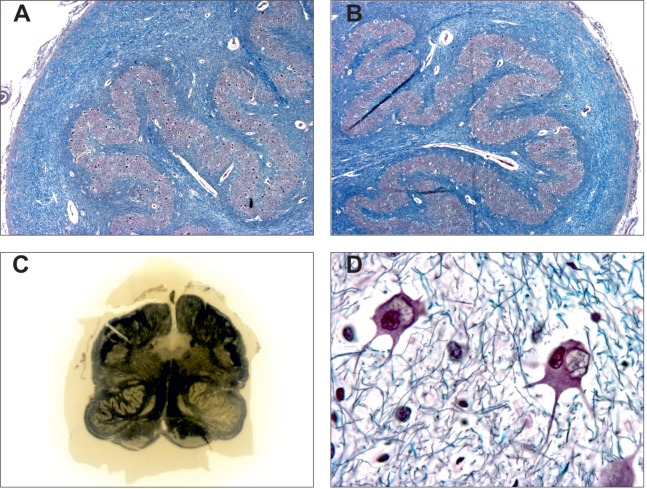
Pathological features of degenerative inferior olive hypertrophy. Hypertrophic inferior olive **(A)** compared to contralateral side **(B)** (Bodian Luxol, X200). Note the mild demyelination of the surrounding white matter. **(C)** Coronal section of the medulla oblongata showing hypertrophy of the left inferior olive (Loyez stain). **(D)** Swelled and vacuolated nerve cells (“fenestrated neurons”) observed in the hypertrophic inferior olive [from **(A)**, Bodian Luxol, X400]. Courtesy of Charles Duyckaerts and Franck Bielle, Escourolle’s Lab, Pitie-Salpetriere Hospital, Paris, France. Adapted from Ref. (51).

Histological features of degenerative olivary hypertrophy had been previously reported by numerous authors, mainly in old French publications (1, 4, 11, 31, 72, 73). On postmortem pathological observations, they described macroscopic hypertrophy of the inferior olives associated with neuron swelling with vacuolation (so-called “fenestrated neurons”), bizarre nerve cell shape, severe fibrillary gliosis, and demyelination of the olive white matter (Figure 3). These pathological hallmarks have been thought to result from transynaptic degeneration secondary to a lesion of the ipsilateral central tegmental tract or the contralateral dentate nucleus. More recent immunohistochemical studies identified various changes in the neurons, their neurites, and presynaptic terminals confirming this hypothesis (74, 75). The main finding is a decreased synaptophysin immunoreactivity confirming the presynaptic abnormalities linked to deafferentation (75). In 1981, Goto and Kaneko published a neuropathological study of eight cases of pontine hemorrhage involving unilaterally or bilaterally central tegmental tracts with different survival periods (76). This study demonstrated six neuropathological stages: (1) no olivary changes (<24 h after onset); (2) degeneration of the olivary amiculum (periphery of the olive, at 2–7 days or more); (3) mild olivary enlargement with neuronal hypertrophy and no glial reaction (at about 3 weeks); (4) culminant hypertrophy of both neurons and astrocytes (at about 8.5 months); (5) olivary pseudohypertrophy with neuronal dissolution (at about 9.5 months and later); and (6) olivary atrophy with neuronal disappearance (after a few years).

Degenerative olivary hypertrophy is predominantly observed in patients with manifest damage of the dentato–olivary pathway (4, 11, 31, 37, 73, 77). It is predominantly but not always associated with PT/OPT, more specifically following head injury (37). All these neuropathological studies agree with the hypothesis of a unique feature of olivary hypertrophy related to transneuronal degeneration in response to deafferentation following dentato–olivary pathway lesion.

Most interestingly, the only pathological study of PAPT with HOD revealed a unique tau pathology (78). This case showed symmetrical unspecific inferior olivary hypertrophy, without focal brainstem lesion. Strikingly, insoluble tau deposits were exclusively found in some infratentorial neurons, in particular in the inferior olives. Combination of primary tauopathy and secondary degenerative changes in the olives suggested to the authors that “primary degenerative process affecting a portion of olivary neurons could trigger retrograde degeneration of the *dentato-olivary* fibers, which might cause secondary (deafferentation type) hypertrophic degeneration in other olivary neurons, perhaps through loss of axon collaterals.” Such a hypothesis of a primary focal tauopathy leading to deafferentation-induced hypertrophic degeneration finds an echo with the observations of HOD in patients with pathologically-proven supranuclear palsy tauopathy (56, 57).

## Radiological Features

### Hypertrophic Olivary Degeneration

**Figure 4:**
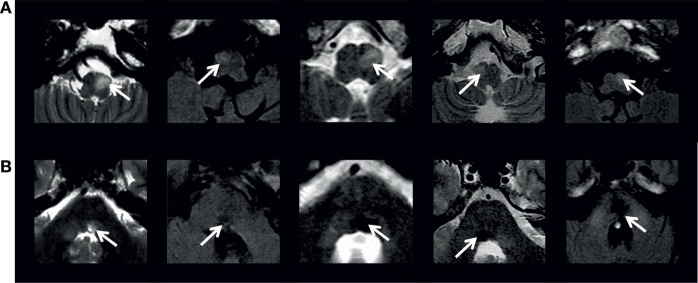
Axial FLAIR or T2 MRI (1.5-T GE scanners) at **(A)** inferior olive level and **(B)** midpontine tegmentum level in five patients with symptomatic oculopalatal tremor. White arrows in **(A)** indicate the abnormal inferior olive hypersignal and in **(B)** the causative lesion. Adapted from Ref. (24).

**Figure 5:**
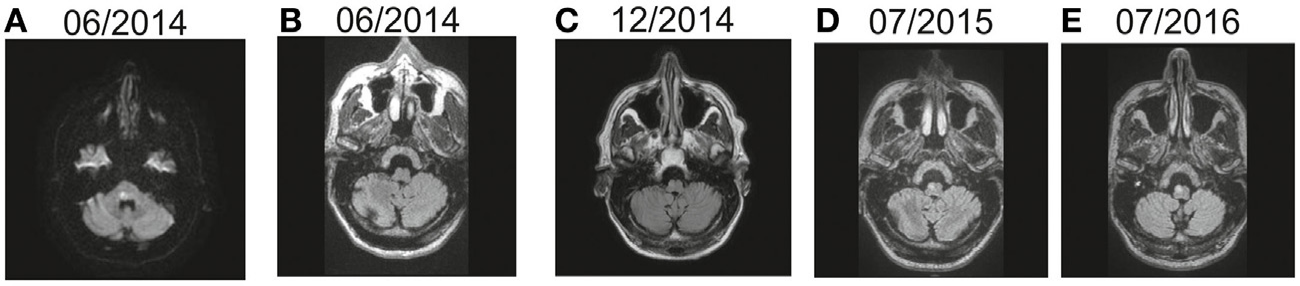
Temporal evolution of right-sided inferior olive hypersignal in a patient with symptomatic oculopalatal tremor. The patient presented a right-sided pontine tegmental lesion in June 2014 seen on the diffusion MRI scan **(A)**, and the medulla showed no abnormal hypersignal on FLAIR MRI **(B)**. Subsequently, right inferior olive hypersignal was observed 6 months later **(C)**, with increasing signal 1 year later **(D)** and right inferior olive hypertrophy was observed 2 years later **(E)**.

The historical observations of neuropathological changes in the inferior olive found their radiological correlates in the observation of increased signal intensity and enlargement of the inferior olive seen on proton density-weighted and T2/FLAIR MRI (6, 7, 79) (Figure 4). The term HOD was then also conventionally used to define these abnormal signals on MRI, even if there is only hypersignal (5, 80). The temporal evolution of these abnormal signals follows pathological changes (81, 82). The hypersignal appears around 1 month after the ictus and persists, while hypertrophy is not usually observed until 6 months after ictus and resolves at approximately 3–4 years after ictus (5) (Figure 5). In some cases, the MRI hypersignal may also return to normal (13, 83). HOD on MRI is unilateral or bilateral in case of symptomatic PT and bilateral in case of PAPT (84, 85). It may also be lacking in familial PAPT (7, 13). In symptomatic PT, HOD usually appears contralateral in case of cerebellar lesion and ipsilateral in case of lateralized central tegmental tract lesion (5, 21, 85). It may precede the clinical manifestations of PT or OPT (80) and even be observed without the development of PT (86). In symptomatic OPT, dissociated pendular nystagmus seems to predict unilateral HOD on MRI with accuracy, while symmetric pendular nystagmus is associated with either unilateral or bilateral HOD (21). Finally, radiological cases of idiopathic HOD without any structural lesion in the Guillain–Mollaret triangle, neither PT, OPT, or PAPT are described (87).

### Cerebral Metabolism Imaging

There is discordance in cerebral metabolism imaging; one study found inferior olive hypermetabolism (88), and the other one that used statistical parametric mapping, failed to show metabolic changes in the inferior olive (34).

### Cerebellar Changes Associated With HOD

MRI of the cerebellum in patients with symptomatic PT found atrophic changes suggesting a degenerative process involving the dentate nucleus and the cerebellar cortex on the side opposite to the HOD (89). Degeneration of cerebellar cortex secondary to HOD has already been discussed in some neuropathological studies (90).

## Physiopathology of PT/OPT Associated with HOD

The main accepted explanation of PT or OPT associated with the development of HOD is that the abnormal inferior olive plays a significant role in PT/OPT (4). First of all, the HOD would develop secondary to dentato–olivary pathway lesion at least for the symptomatic forms, due to a denervation mechanism (77). Normal inferior olivary neurons can generate spontaneous oscillations and are electrically coupled by dendrodendritic gap junctions (91, 92). In case of dentato–olivary pathway lesion, denervated olivary neurons released from inhibitory inputs would enlarge and develop sustained synchronized oscillations (91). Animal models of HOD show the development of spikes on denervated inferior olivary neurons, supporting electrotonic coupling through gap junctions (93). In this hypothesis, inferior olive would be the oscillator of palatal and/or ocular tremor. This is further supported by the observation of disturbed cerebellar function (motor learning) in patients with SPT (94, 95) and the temporal relationship of the development of HOD and the clinical symptoms. This is finally further supported by the observation of inferior olivary nucleus hypermetabolism (86). However, the main criticism against the involvement of inferior olive as part of the mechanism for OPT is the observation of decreased hypertrophy of inferior olive in time while OPT persists, or other observations showing absent inferior olivary nucleus hypermetabolism (34) in patients, and functional imaging showing synchronous decreased cerebellar activity and OPT with clonazepam, but no decrease of inferior olive activity (96). Some authors suggested that inferior olive could be involved in the development of PT/OPT but not in maintaining the symptoms (35).

**Figure 6:**
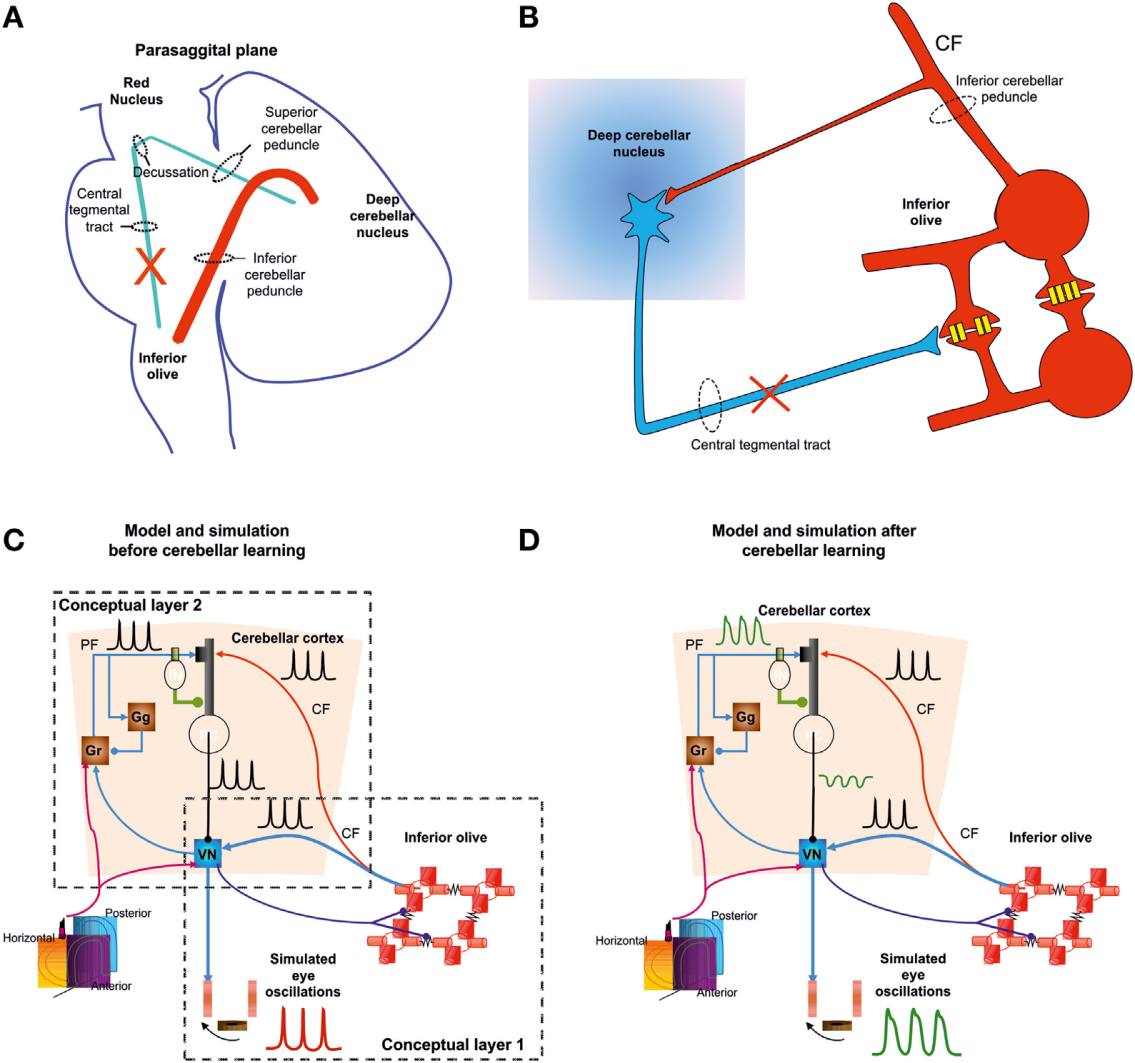
Schematic representation of the Guillain–Mollaret triangle formed by connections between the deep cerebellar nuclei and contralateral inferior olive, which pass near the red nucleus **(A)**. The conduction strength through the dendrodendritic gap junctions (schematized with yellow connexon channels; DD) between adjacent inferior olivary neurons are inhibited by projections from the deep cerebellar nuclei (blue projection) **(B)**. Lesions in the Guillain–Mollaret triangle [red X in **(A,B)**] also result in hypertrophy of inferior olive neurons causing development of abnormal soma-somatic gap junction. Schematic representation of a model for classical delay conditioning **(C,D)**. Model and traces from simulations after inferior olive hypertrophy but before cerebellar learning **(C)**. Inferior olive and cerebellar modules after hypertrophy and learning **(D)**. Lower left corner shows icon for semicircular canals **(C,D)**. Simulated membrane potentials (black), eye oscillations (magenta). CF, climbing fibers; PF, parallel fibers; DD, dendrodendritic gap junction; SS, soma-somatic gap junction; Gr, granule cell layer; IN, interneurons; PC, Purkinje neurons [(27) with permission for reproduction of material].

A fascinating recent model suggested both the implication of inferior olive oscillator generating spike trains at 1–2 Hz and cerebellar modulation/amplification of the motor output (27, 97) (Figure 6). In this model of pendular nystagmus in OPT, the synchronized signal from the inferior olive reaches *via* climbing fibers Purkinje cells and the deep cerebellar nuclei including vestibular nuclei. In turn, the signal in the vestibular nuclei projects indirectly to the Purkinje cells, *via* a mossy fiber/granule cells-parallel fiber. The repeated inferior olive pulses would create periodic climbing and parallel fiber inputs to Purkinje cells at approximately the same time and create a learning signal back to the vestibular nuclei, contributing to smoothing and amplifying pulse (27). While this model seems to reproduce many of the aspects of OPT and specifically the 1–2 Hz irregular oscillation, it cannot prove that both inferior olive and cerebellum are necessary to explain it.

The topography of this tremor involving structures corresponding embryologically to the first to fifth branchial arches has received less interest. In 1949, Stern suggested that PT would be the human homolog of a primitive accessory respiratory reflex in gill-breathing vertebrates, leading to the hypothesis of recurrence of an archaic phenomenon (98). The limitation to the branchial arches muscles suggested to authors that the central tegmental tract lesion causes hypersensitivity of the nucleus ambiguous that innervate branchial muscles (99). However, this does not properly explain how 1–2 Hz oscillation develop in the arches, while Shaikh’s model does.

## Treatment

**Figure 7:**
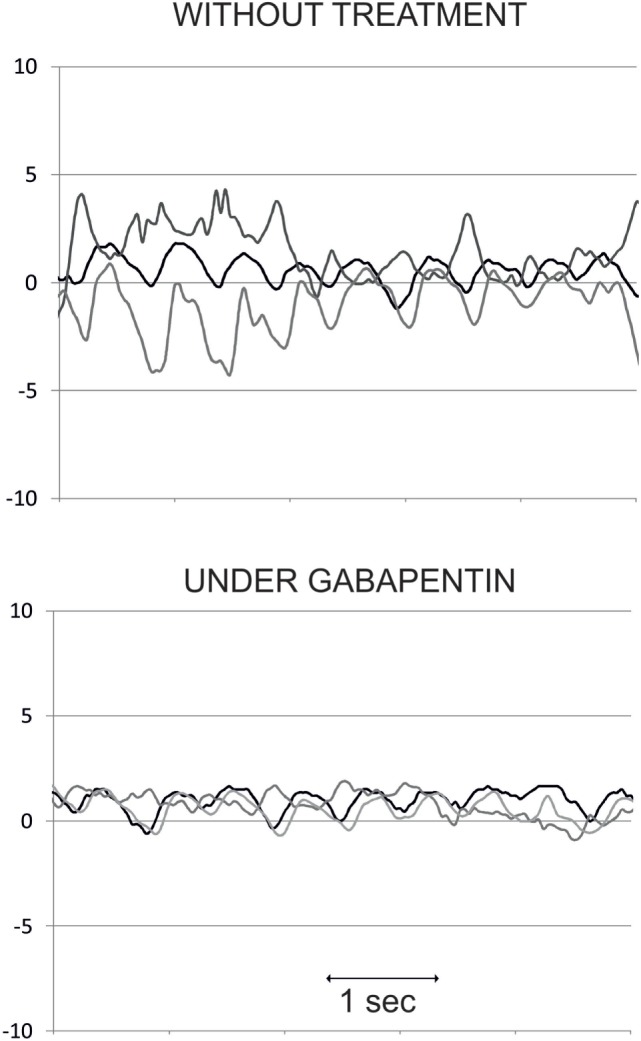
Eye position (in degrees) traces over time (in seconds) in one oculopalatal tremor patient, without treatment (upper panel) and under gabapentin (lower panel). Dark line: horizontal position, gray line: vertical position, and light gray line: torsional position. Note the decrease in nystagmus amplitude, mainly in the torsional plane, under gabapentin.

Therapeutic trials have been mainly performed on acquired pendular nystagmus, which is the most symptomatic consequence of OPT. The most rigorous treatment trials in acquired pendular nystagmus (due to MS or OPT) led to the proposal of gabapentin or memantine as valuable drugs (26, 100–104). Only one study specifically tested gabapentin, memantine, and baclofen in a group of six patients with acquired pendular nystagmus in OPT with a significant effect of gabapentin and memantine on reduction of nystagmus amplitude and frequency irregularity (102). We have also observed sustained decrease of nystagmus velocity in some patients (Figure 7). Another study found marked improvement of both eye and palate movements as well as complaints by patients (including audible clicks) with trihexyphenidyl; however, patients with PT following a structural lesion, EPT, and MS, but not those with OPT, were included (105). There have been suggestions of testing drugs that reduce electrotonic coupling among hypertrophied inferior olive neurons by blocking connexons like quinine, carbenoxolone, or mefloquine (27), but no study has since been published. Botulinum toxin has been tested on pendular nystagmus in OPT with variable success (106) and in clicking tinnitus in PT (107).

In a different approach, bilateral deep brain stimulation of the red nucleus in one patient with OPT (and failure of medical treatment) was tested (108). This study failed to show any improvement of eye oscillation. The failure of this intervention may be explained by erroneous interpretation of mechanism of OPT. The hypothesis was to interfere with the rhythmicity of the olivocerebellar circuit, but the target was the afferent dentato–olivary pathway within the red nucleus region.

## Author Contributions

Both CT and VD contributed to conception or design of the work; drafting the work and revising; final approval of the version to be published; and the agreement to be accountable for all aspects of the work in ensuring that questions related to the accuracy or integrity of any part of the work are appropriately investigated and resolved.

## Conflict of Interest Statement

The authors declare that the research was conducted in the absence of any commercial or financial relationships that could be construed as a potential conflict of interest. The reviewer MW and handling Editor declared their shared affiliation and the handling Editor states that the process nevertheless met the standards of a fair and objective review.

## Supplementary Material

Video S1 Palatal and chin tremor. This 30-year-old patient presented an acute pontine hemorrhagic stroke resulting in left hemiplegia and right facial palsy. Three months later, he complained of oscillopsia. T2 MRI showed pontine hemorrhagic scar. In addition to pendular nystagmus (not shown), synchronous left-sided palatal and chin tremor around 1 Hz frequency can be observed in this video. T2 MRI showed left-sided hypertrophic olivary degeneration at medullar level.

Video S2 Ocular and upper lip tremor. This 29-year-old patient presented an acute hemorrhagic pontine stroke. He presented with right-sided hemiplegia, left-sided facial palsy, dysarthria, and left-sided gaze palsy. A few months later, he complained of oscillopsia. The video shows binocular incongruent 1.6 Hz pendular nystagmus, and left upper lip synchronous tremor. There was also palatal tremor (not shown). MRI found left-sided hypertrophic olivary degeneration.

Video S3 Decrease of pendular nystagmus with time in a patient with symptomatic oculopalatal tremor (OPT). OPT was diagnosed in 2012 but occurred a few weeks after surgical treatment of bleeding brainstem cavernoma in 2010. During follow-up, the patient described a decrease in oscillopsia and observation disclosed great decrease in nystagmus in 2016, without any pharmacological treatment.

## Abbreviations

HOD, hypertrophic olivary degeneration; PT, palatal tremor; OPT, oculopalatal tremor; PAPT, progressive ataxia and palatal tremor; EPT, essential palatal tremor.
